# Adsorption and separation of Cs(I) and Ba(II) from aqueous solution using zinc ferrite-humic acid nanocomposite

**DOI:** 10.1038/s41598-023-32996-5

**Published:** 2023-04-11

**Authors:** M. I. A. Abdel Maksoud, G. A. Murad, W. F. Zaher, H. S. Hassan

**Affiliations:** 1 Radiation Physics Department, National Center for Radiation Research and Technology (NCRRT), Egyptian Atomic Energy Authority (EAEA), Cairo, Egypt; 2 Hot Laboratory Center, Egyptian Atomic Energy Authority (EAEA), P.O.13759, Cairo, Inshas Egypt

**Keywords:** Environmental sciences, Materials science, Nanoscience and technology

## Abstract

Reclaimable adsorbents have an essential role in removing radionuclides from waste streams. Herein, zinc ferrite-humic acid ZFO/HA nanocomposite was synthesized for effective cesium and barium adsorption. The prepared ZFO/HA nanocomposite was analyzed using analytical techniques including XRD, FTIR, EDX, and SEM. From kinetic studies, the mechanism adsorption process follows the second model. The isotherm studies clarified that the Langmuir model fit the adsorption of both ions onto the prepared sample, and the monolayer capacities are equal to 63.33 mg/g and 42.55 mg/g for Ba(II) and Cs(I), respectively. The temperature parameter was also studied, and the adsorption reaction was spontaneous and endothermic. The maximum separation between two ions was achieved at pH 5 (αCs/Ba = 3.3).

## Introduction

The development of nuclear applications has led to the establishment of nearly 400 power plants working in various countries. These power plants generate about twelve thousand tons of radioactive waste annually^1,2^. Increasing nuclear power plant use is bound to the efficient, safe procedure of nuclear reactors and fuel cycle operation. Nevertheless, no effective method was found to minimize confusion when this leak occurs from these power plants. Several nuclear accidents occur in different countries, such as in Chornobyl and Fukushima; these accidents cause the liberation of different radionuclides, such as ^90^Sr, ^137^Cs, ^79^Se, and ^129^I, into the environment^3–5^.

Nuclear power plants' operation gives rise to releasing several radioactive isotopes that must be significantly handled to keep safe human health and the surrounding environment. Different techniques should be improved to remove these isotopes from the surrounding environment to treat this significant issue. Several methods, such as filtration, precipitation, ion exchange, adsorption, and reverse osmosis, are applied to eliminate radioactive isotopes from radioactive waste. The radioactive waste treatment based on the adsorption/ion exchange technique remarkably removes several radioisotopes from the waste stream. Also, this method has many applications in handling different types of waste. So, the ion exchange technique has become almost promising^6–12^. Natural exchanger materials, such as zeolites, different types of clay, and oxide materials, have been investigated and applied to remove radioactive wastes. Also, synthetic exchangers such as composite materials, polymeric materials, and porous materials have been of greatest interest for removing wastes^9–11,13–17^.

Magnetic adsorbents get the feature of being efficiently recovered from the aqueous system by utilizing a magnetic field^18–26^. Spinel ferrites possess unique chemical and physical features that distinguish them from their bulk form; hence they are often utilized as adsorbents. Spinel ferrites demonstrate long-term effectiveness in wastewater treatment because of unique characteristics such as uneven surface, a large surface-to-volume ratio, customizable size, and remarkably magnetic properties^27,28^. Among spinel ferrites, ZnFe_2_O_4_ nanoparticles, ZFO NPs, is one significant composite utilized in many promising applications, including biosensors, photocatalysts, magnetic fluids, and rechargeable battery materials. Different characteristics of ZFO NPs, such as non-toxicity, strong phase resistance, cheap cost, visible light absorption, and insoluble in water as well as moderate corrosion resistance, make these materials employed in water treatment technologies to eliminate different dyes and hazardous materials^29,30^. H. Hassan et al.^27^ have reported an investigation into the effectiveness of ZFO NPs in the elimination of radionuclides (Cs and Eu) from a solution of nitric acid.

Humic acid (HA), a natural organic matter (NOM) fraction, is an attractive material for decorating metal oxides that might be used in an environmentally friendly technique. The HA has the highest proportion of carboxyl and phenolic hydroxyl groups responsible for electrostatic interactions, ion exchange, redox, chelation, and a higher prospect for anion and cation sorption. Rashid et al.^31^ have reported that HA-coated Fe_3_O_4_ removes toxic phosphate from aqueous media. Recently, Xue et al.^32^ used HA-modified Fe_3_O_4_ to absorb heavy metals from water.

The removal of hazardous ion (Ba(II) and Cs(I)) from aqueous solution using synthesized zinc ferrite-humic acid nanocomposite as exchanger material has never been reported in the literature. This study aims to prepare the ZFO-HA composite and investigate it as adsorbent material to remove Cs(I) and Ba(II) from the aqueous solutions. Several analytical techniques have been applied to depict the prepared ZFO-HA. Various parameters, such as the effect of pH values, time, the effect of initial concentration, and temperature, were studied to specify the adsorption process.

## Experimental

### Materials

All used reagents in this work are analytical grade and utilized without purification. All of the investigations employed double-distilled water. Iron (III) nitrate nonahydrate (Fe(NO_3_)_3_·9H_2_O), zinc nitrate hexahydrate (Zn(NO_3_)_2_·6H_2_O), and citric acid monohydrate (C_6_H_8_O_7_.H_2_O). Cesium or barium solutions have been produced in the experiments via dissolved CsCl or Ba(NO_3_)_2_.7H_2_O into double-distilled water.

### Synthesis of zinc ferrite ZFO

The synthesis of ZFO NPs was synthesized via the sol–gel method by using iron (III) nitrate nonahydrate (Fe(NO_3_)_3_·9H_2_O) and zinc nitrate hexahydrate (Zn(NO_3_)_2_·6H_2_O) as sources for Fe and Zn, respectively. Firstly, a stoichiometric ratio of Fe: Zn nitrate compounds (2:1) was separately dissolved in 50 ml of distilled water. The two solutions were now combined and stirred for 15 min. A 50 ml of 3 M citric acid monohydrate solution was added as an efficient and environmentally friendly fuel to the present solution at 70 °C with a vigorous stirrer for 45 min. The resultant solution is heated to 120 °C for two h to assist gel formation. The resultant gels were again dried and ground to produce ZFO powder. Lastly, the powder is sintered for 120 min at 500 °C^27^.

### Synthesis of zinc ferrite-humic acid composite

The natural humic acid (HA) used in this work was isolated from the agricultural land soil of (Ibri, Oman). The form of HA used is sodium humate, the characterization of HA is displayed in detail in the previous study ^6^. The HA solution was prepared using a certain weight of HA (0.03 g) dissolved in a specified volume from 0.1 M NaOH (50 mL); the solution was stirred for about one hour under the steam of nitrogen gas; this evaded air oxidation. Titration of the solution with 0.1 M HCl was done until certain pH values (7.5 ± 0.1). The solution was diluted to about 100 ± 2 mL until the acquired concentration (CHA_i_) became 300 ± 15 mg/L. ZFO NPs with HA were prepared using the following steps, about 2.043 g of ZFO NPs was mixed with about 50 ± 1 mL of the prepared HA solution. Then the solution of HA with ZFO NPs was equilibrated to about 48 h at 25 °C. The solution was centrifuged for about 8 min at 600 rpm. The obtained precipitate was separated and collected. The precipitate was washed in distilled water about three times; then, UV-spectrophotometry measured supernatants to display no free HA liberated from the Z (< 0.05%). The solid phase was dried by freezing, and then the solid phase was stored at 25 °C to start using. A calibration curve was plotted to the standard humic acid solution, and the remaining concentration of humic acid was measured (CHA_fi_) in the supernatant by determining absorbance for the initial ( CHA_i_ ~ 15 mg), also the residual humic acid solution by 160A UV–visible spectrometer, Shimadzu, Japan, at wave number equal 420 nm and absorption cell (10 mm) from quartz^6^. The amount of loaded humic acid in the ZFO NPs has been computed by deducting CHA_fi_ ~ 0 mg (final concentration) from the initial concentration (CHA_i_ − CHA_fi_). The final mass ratio in ZFO/HA composite was about 15 mg^33^.

### Instruments and apparatus

Various tools were performed to evaluate the various physical characteristics of the ZFO/HA composite. The ZFO/HA composite's surface morphology was achieved using a scanning electron microscope, model JSM-6510A from Japan. The XRD measurements were conducted by an X-ray diffractometer purchased from XRD 6000, Shimadzu, Japan. EDX was used to inspect the chemical composition of the ZFO/HA composite. Nicolet spectrometer, Meslo, USA, was used to capture the FTIR spectrum.

### Sorption studies

Sorption experiments have been done to estimate the elimination of the cesium and barium ions. These experiments were achieved in the shaker at room temperature to research the effect of pH values and shaking time and cesium and barium concentration ion effect on the sorption behavior of the ZFO/HA composite. Each vial comprised 0.01 g of the ZFO/HA composite with a volume of cesium and barium ions solution (10 mL). Both ions' initial concentrations (C_0_) vary from 100 to 350 mg/L. All vials have been sealed and shaken to the equilibrium case. The loaded material was separated from the rest of the solution using centrifugation. The sorption studies were done at temperatures ranging from 25 to 55 °C. In these studies, an exemplary shaking was executed employing a thermostatic shaker, model Julabo SW-20 C Germany. The pH has been controlled by 0.1 M of NaOH or HCl. The removal, *%R*, as well as the amount of ions adsorbed, *q*_*t*_, mg^−1^ g, were counted using the next relations respectively^34^:

1 $$\user2{\% R} = \frac{{{\varvec{C}}_{{\varvec{o}}} - {\varvec{C}}_{{\varvec{t}}} }}{{{\varvec{C}}_{{\varvec{o}}} }} \times 100$$

2 $${\varvec{q}}_{{\varvec{t}}} = \frac{{\user2{\% R}}}{100} \times {\varvec{C}}_{{\varvec{o}}} \times \frac{{\varvec{V}}}{{\varvec{m}}}$$

3 $${\varvec{K}}_{{\varvec{d}}} = \frac{{{\varvec{C}}_{{\varvec{o}}} - {\varvec{C}}_{{\varvec{e}}} }}{{{\varvec{C}}_{{\varvec{e}}} }} \times \frac{{\varvec{V}}}{{\varvec{m}}}$$

where C_o_ and C_t_, mg/L, point out the initial and final concentration for the solution at the beginning and end of the sorption process, respectively, V refers to the solution volume, L, as well as m, g, symbolizes the mass of ZFO/HA composite, k_d_ represented distribution coefficient for studied ions.

### Separation factor

Separation factor (α) was applied to investigate the capability of two ions to be adsorbed onto prepared sorbent materials from solutions that equal concentration. The separation factor of two ions can be calculated from the ratio of the distribution coefficient of each ion, as shown in the following:

4 $$\alpha_{B}^{A} = \frac{{K_{dA} }}{{K_{dB} }}$$

K_dA_ and K_dB_ represent the distribution coefficients of the two ions.

### Desorption studies

The recovery of Ba(II) and Cs(I) loaded on the ZFO/HA nanocomposite adsorbent was achieved using different reagents such as HNO_3_, HCl, H_2_SO_4_, and NaOH.

### Modeling of cesium and barium ions

#### Kinetic modeling

The sorption kinetics of cesium and barium ions using the ZFO/HA nanocomposite were studied employing various kinetic models such as the pseudo-first and pseudo-second order^35,36^. The equation of the pseudo-first model is written as follows:

5 $$\log \left( {q_{e} - q_{t} } \right) = \log q_{e} - \left( {\frac{{k_{1} }}{2.303}} \right)t$$

where qt symbolizes the amount of Cs(I) and Ba(II), mg^-1^ g, adsorbed onto prepared ZFO/HA nanocomposite at any time t, while the adsorbed amount of ions, q_e_ mg^-1^ g, at equilibrium as well as k_1_ is the constant of this model, min^-1^, the straight line obtained from the relation between log (q_e_ − q_t_) with t suggests that this sorption process can be obeyed to this model. Values of q_e_ and k_1_, were computed from intercept and slope for linear relation, respectively. While the pseudo-second model has been displayed as the following equation:

6 $$\frac{t}{{q_{t} }} = \frac{1}{{k_{2} q_{e}^{2} }} + \frac{1}{{q_{e} }}t$$

The k_2_ represents this model's constant (g/mg.min) by plotting the relation between t/qt with time, t. The straight line has been acquired, explained that this model may be applicable. The constant of this model (qe and k_2_) was determined from the slope and the intercept of this relation, respectively.

#### Sorption isotherm studies

Two isotherm models have been employed to investigate the adsorption behavior. The Langmuir, as well as Freundlich model, was applied. The impact of carrier concentration on the adsorption process for Cs(I) and Ba(II) was studied at the concentrations range (of 100 to 350 µg/mL). The linear form of the Langmuir model can be described as illustrated in the following equation^3^:

7 $$\frac{{C_{e} }}{{q_{e} }} = \frac{1}{{bQ_{\max } }} + \frac{1}{{Q_{\max } }} \times C_{e}$$

C_e_ refers to the concentration of ions at equilibrium (mgL^−1^), while q_e_ symbolizes the amount of studied ions (mg/g) in the equilibrium case. In contrast, the Q^max^ refers to the capacity of the adsorption process (mg.g^-1^), and b represents the constant of the Langmuir equation regarding the energy of adsorption. Plotting a relation between (C_e_/q_e_) and Ce gives linear relation. The constants of this model can be determined from this line (Q_max_ and b). The intrinsic feature of this model was revealed in terms of constant (R_Ld_), which is written as follows^37^:

8 $$R_{Ld} = \frac{1}{{1 + bC_{o} }}$$

b points out a constant of this model, and C_o_ refers to the concentration of both ions. R_Ld_ values signalized the nature of isotherm, favorable when 0 < R_Ld_ < 1, irreversible if R_Ld_ = 0, but unfavorable R_Ld_ > 1, or linear if R_Ld_ = 1^37^.

Equation of the Freundlich model count on the adsorption process onto the heterogeneous surface and can be displayed as follows^8^:

9 $$\log q_{e} = \log k_{f} + \frac{1}{n}\log C_{e}$$

K_f_ and n symbolize to amount adsorbed of ion and the intensity of adsorption, respectively.

### Thermodynamic studies

The parameters of thermodynamic studies (ΔG◦, ΔH◦, and ΔS^o^) to the adsorption process for Cs(I) and Ba(II) toward ZFO/HA composite were studied using the following equations^36^:

10 $$\Delta G^{{\text{o}}} = - RT\ln K_{C}$$

11 $$K_{C} = \frac{{q_{e} }}{{C_{e} }}$$

12 $$\ln \,K_{C} = \frac{{\Delta S^{{\text{o}}} }}{\,R} + \frac{{ - \Delta H^{{\text{o}}} }}{R}\frac{1}{T}$$

The equilibrium constant symbolizes Kc, the general gas constant, R, and the temperature is T (K). The straight curve was acquired from the plot between the relation Kc against 1/T. ΔH, as well as ΔS°, was evaluated from the slope and intercept of this relation, respectively.

## Results and discussion

### Characterization of the synthetic by zinc ferrite-humic acid composite.

**Figure 1:**
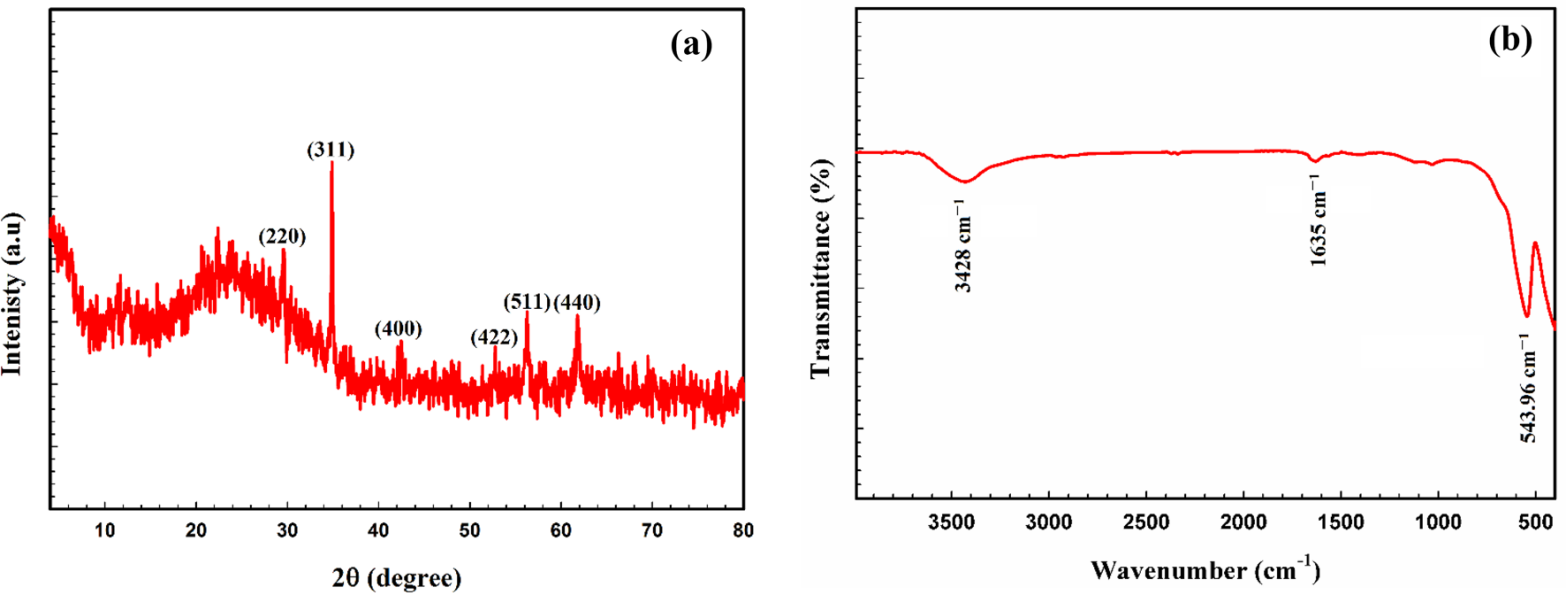
(a) XRD and (b) FTIR spectra of ZFO/HA nanocomposite.

Figure 1a depicts the XRD pattern of the ZFO/HA nanocomposite. The reflection planes (220), (311), (400), (422), (511), and (440) in the pattern reveal the presence of single-phase ZFO NPs with a face-centered cubic spinel structure, as presented in previous work^27^. This result demonstrated that the crystal structure of ZFO NPs does not change after HA modification. The crystal size of the ZFO/HA nanocomposite was determined via Scherrer’s equation^38^:

13 $${\varvec{D}} = \user2{ }\frac{{0.9\user2{ } \times {\varvec{\lambda}}}}{{{\varvec{\beta}}_{{{\varvec{FWHM}}}} \user2{ } \times \cos {\varvec{\theta}}}}$$

${\upbeta }_{\mathrm{FWHM}}$ is the full-width at half maximum, (λ = 0.154178 nm), and *θ* is the Bragg angle. The results showed that the ZFO/HA nanocomposite has a crystallite size of 61 nm.

Figure 1b shows the FTIR spectra of the ZFO/HA nanocomposite. Spinel ferrites possess two distinct vibrational bands related to the stretching vibration of tetrahedral groups (A-site) and octahedral groups (B-site). For the ZFO/HA nanocomposite, the band at υ_1_ = 543.96 cm^−1^ refers to the A-site stretching in ZFO NPs, demonstrating that the cubic spinel phase was successfully prepared for the ZFO/HA nanocomposite. While the O–H stretching facilitated identifying the HA functional group at the 3428 cm^−1^. The absorption band at 1635 cm^−1^ was attributed to aromatic C = C stretching^32^. XRD data was matched well with FTIR results, confirming the successful synthesis of the ZFO/HA nanocomposite.

**Figure 2:**
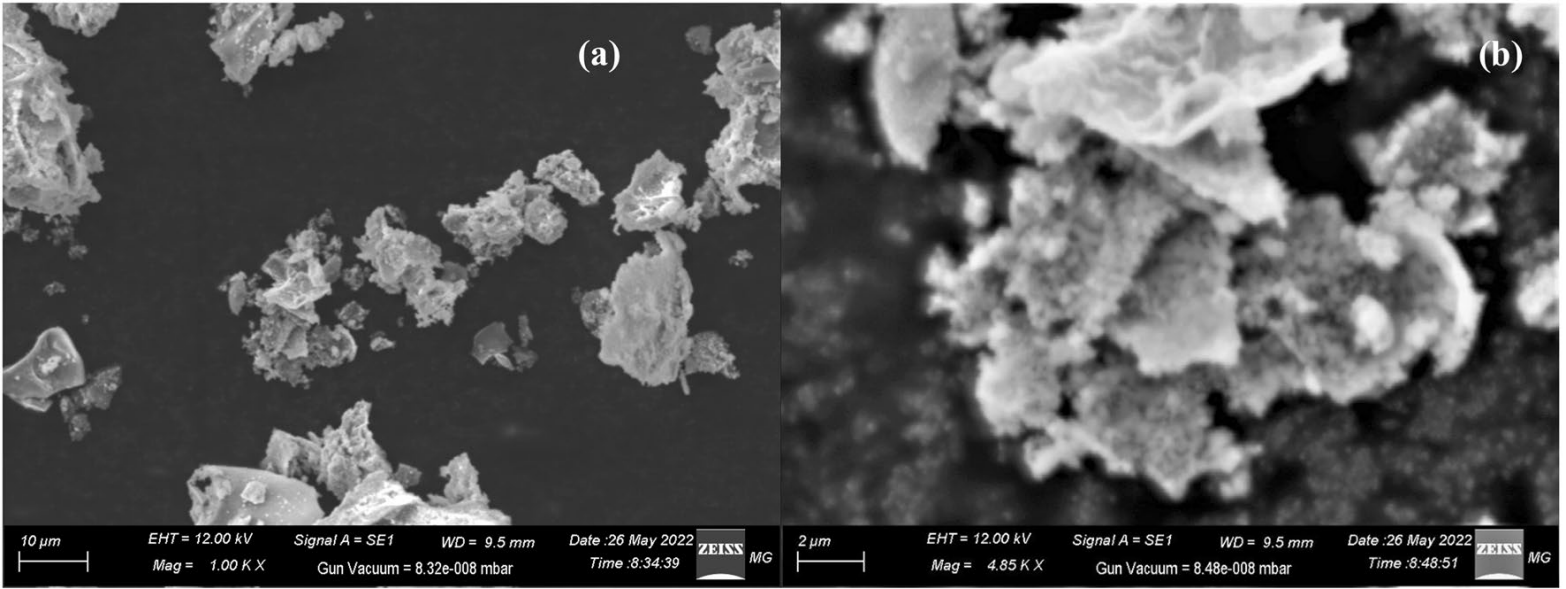
(a&b) SEM micrographs of the ZFO/HA nanocomposite.

SEM determined the surface morphology of the ZFO/HA nanocomposite, as seen in Fig. 2 (a&b). The figure illustrated that the ZFO/HA nanocomposite has a flake-like shape. Also, the figure revealed the grain size distribution for the ZFO/HA nanocomposite in the nanoscale range. In addition, the figure shows a non-uniform distribution of agglomerated particles with a high amount of pores.

**Figure 3:**
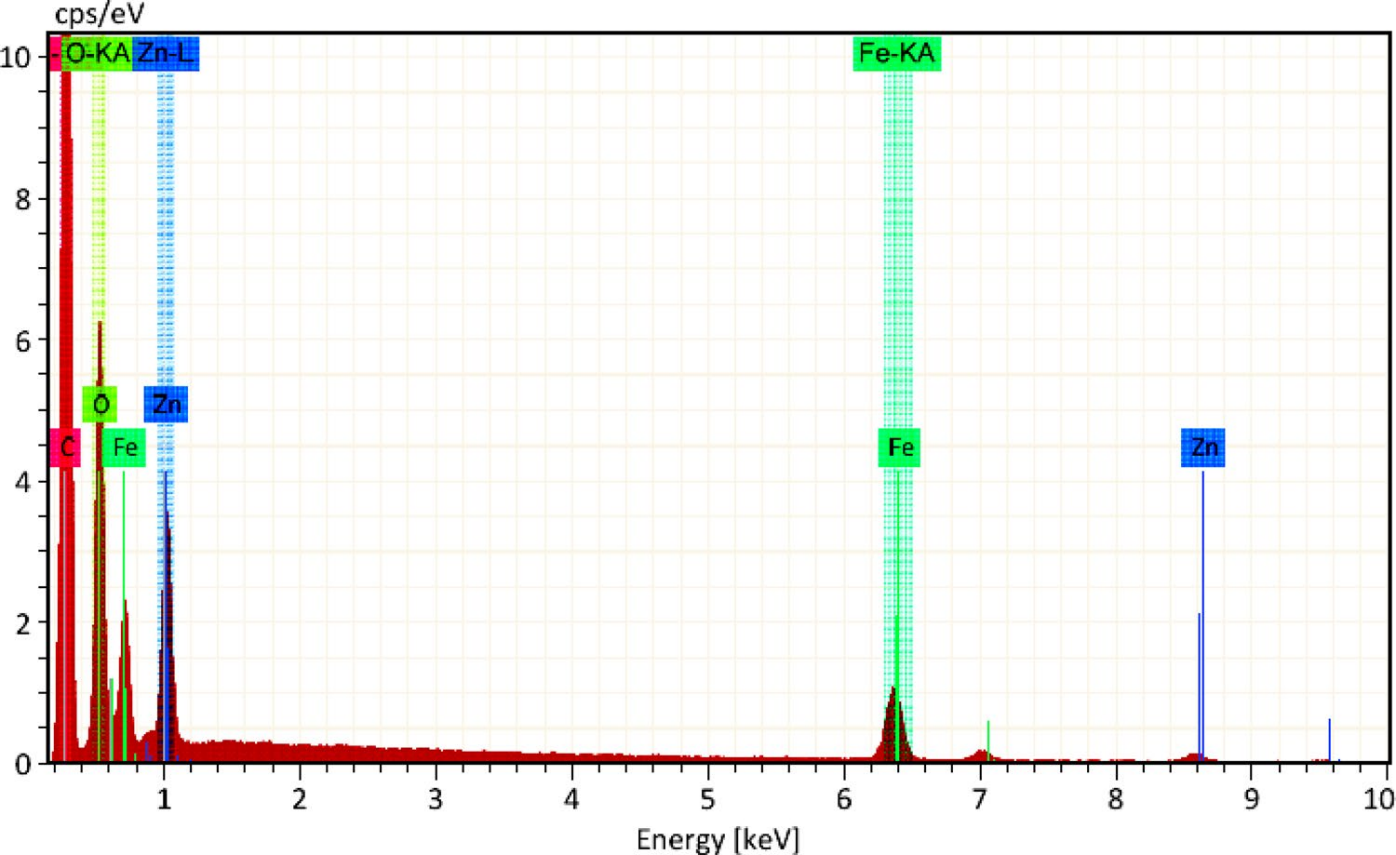
EDX spectra of the ZFO/HA nanocomposite.

**Figure 4:**
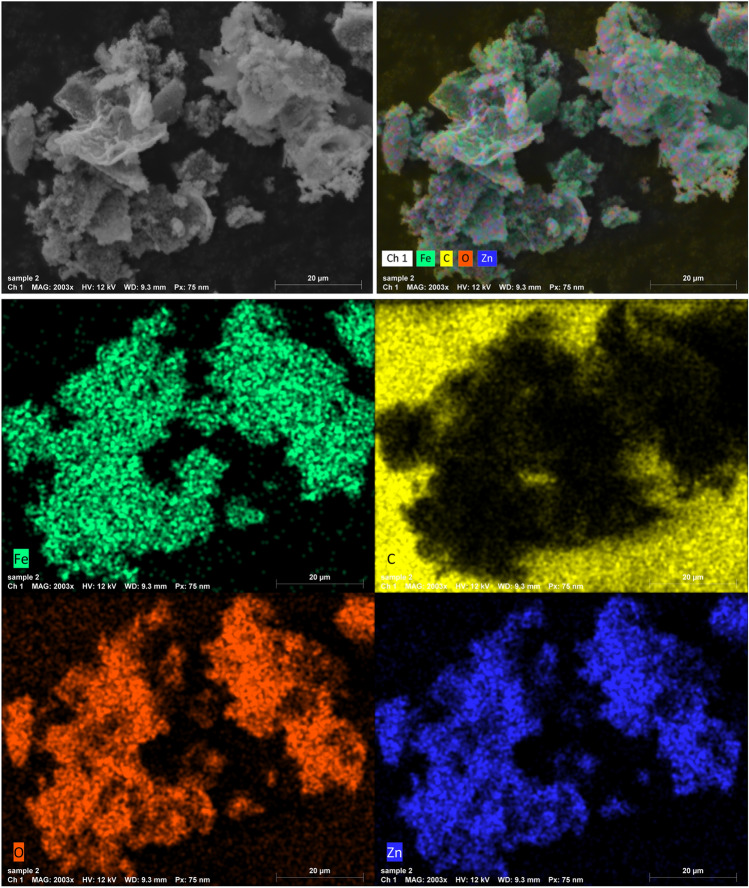
Mapping images of ZFO/HA nanocomposite.

The EDX technology offers extensive characteristics for determining the sample's composition and mapping its components. The EDX spectra of the ZFO/HA nanocomposite are illustrated in Fig. 3. Clearly, C, Zn, O, and Fe are involved in stoichiometric proportions without any foreign elements, which proves the purity of the ZFO/HA nanocomposite. In addition, the mapping images demonstrated that all whole elements are uniformly distributed throughout the ZFO/HA nanocomposite, see Fig. 4.

### Cs(I) and Ba(II) adsorption

#### Effect of pH

**Figure 5:**
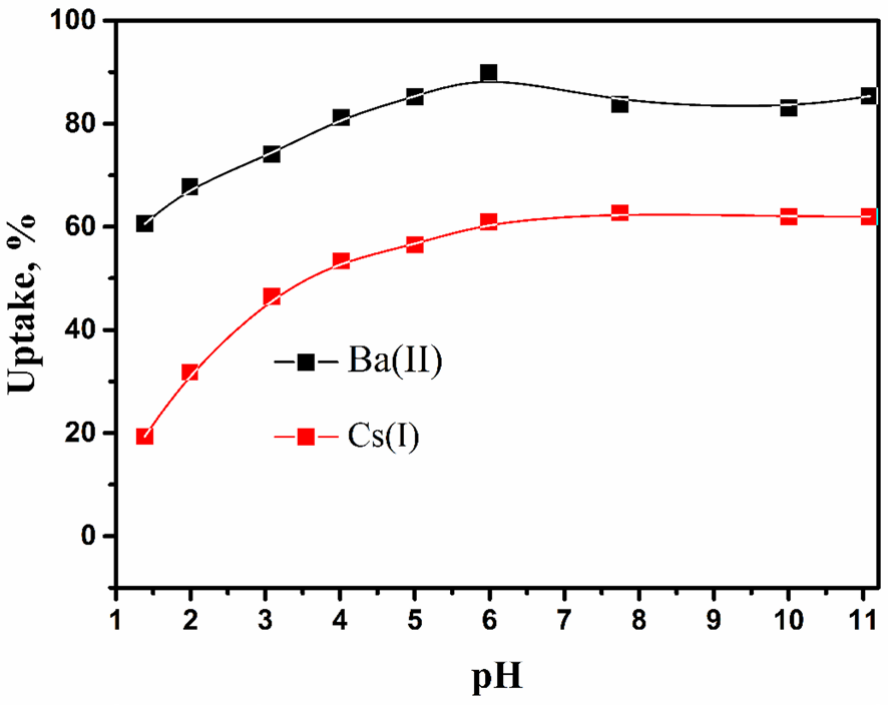
Effect of pH on the sorption of Cs(I) and Ba(II)by ZFO/HA nanocomposite.

Different pH values have remarkably affected the adsorption behavior of different ions from the waste stream^39^. The pH effect on adsorption behavior has been estimated. Figure 5 displays the impact of pH values on the uptake of Cs(I) and Ba(II) in the range of pH 1 to 11 at a certain concentration of Ba(II) and Cs(I) (C_o_ = 100 mg/L) with shaking overnight at room temperature. The figure shows, with increasing pH values, uptake for both ions increases. Also, it is noted that maximum uptake has been attained by nearly pH 5 for each ion. The optimal acidity for Cs(I) and Ba(II) uptake has reached a pH of 5. Thus, a pH of 5 has been selected to remove each ion in all following experiments.

#### Impact of shaking time

**Figure 6:**
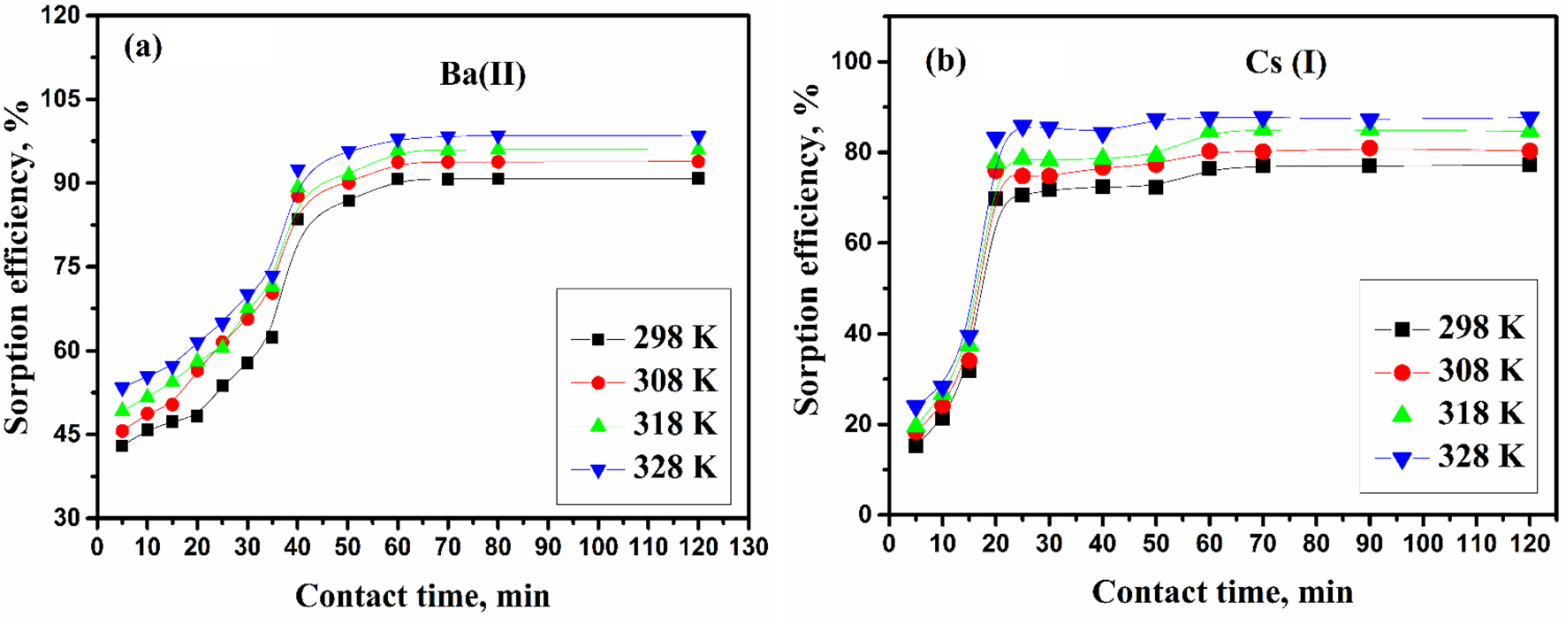
impact of shaking time on the sorption of (**A**) Ba(II) and (**B**) Cs(I) by ZFO/HA nanocomposite.

Influencing the shaking time on cesium and barium ions adsorption onto ZFO/HA nanocomposite at various temperatures is displayed in Fig. 6. It is evident that fast initial sorption for Cs(I) and Ba(II) execute at the onset of shaking time, and so sorption behavior of studied ions increases as time rising. The rate of each ions adsorption is greater at the beginning; this may be due to the high surface area of the ZFO/HA nanocomposite in this study; the uptake of Ba(II) is 90.7% while 76.2% is the uptake of Cs(I). The uptake was achieved at 60 min. Moreover, no notable diversity in the adsorption behavior of both ions after 120 min.

The amount of Ba(II) and Cs(I) adsorbed onto ZFO/HA nanocomposite increases as the temperature increases. The rising temperature raises the diffusion rate of studied ions onto the superficial boundary layer and interior pores for ZFO/HA nanocomposite. Also, As the temperature increases, this may cause the swelling of the internal structure of the ZFO/HA nanocomposite-authorized ions to penetrate further ^34^.

#### Adsorption kinetic studies

The kinetic experiments of the sorption process are significant because these studies supply helpful information about the adsorption mechanism. The kinetic studies for Ba(II) and Cs(I) onto ZFO/HA nanocomposite were achieved using two kinetic models. Pseudo-first-order, as well as pseudo-second-order models, were applied.

##### Pseudo-first-order

**Figure 7:**
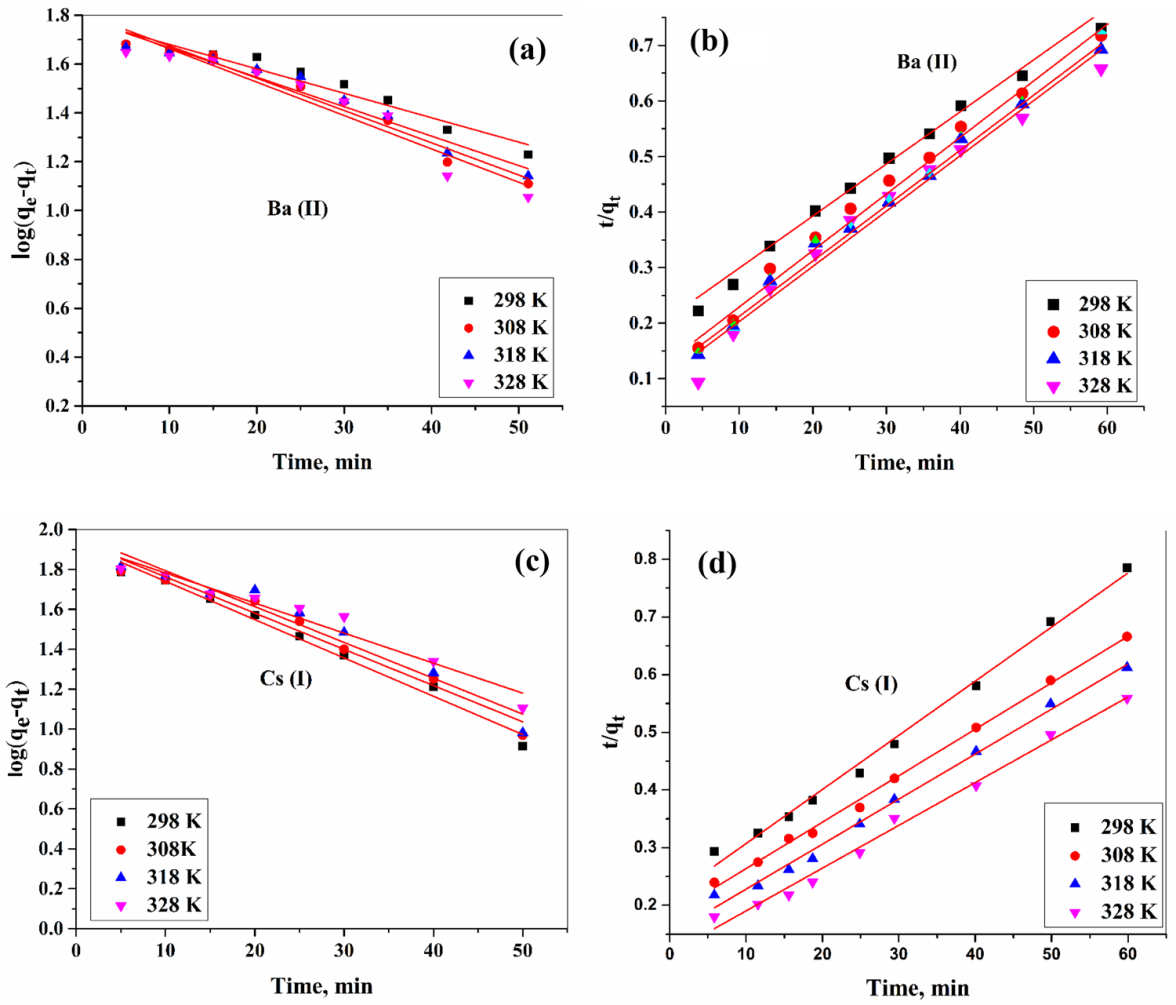
Pseudo-first-order (**a**, **c**) and Pseudo -second-order (**b**, **d**) plots for the adsorption of Ba(II) and Cs(I) by ZFO/HA nanocomposite.

Data displayed in Table 1 and Fig. 7 was shown that the amount of ions adsorbed (q_cal_) onto ZFO/HA nanocomposite differ considerably from those estimated experimentally (q_exp_), proposing that the sorption behavior for Ba(II) as well as Cs(I) is not following first-order- model.

**Table 1:** The calculated parameters of the kinetic models of **Ba(II)** and **Cs(I)** for adsorption onto ZFO/HA nanocomposite at different temperatures.

Ion	Temp.,* K*	Pseudo-first-order Parameter			Pseudo-second-order parameter			*q *_*exp*_* (mg/g)*
		*k*_*1*_*, (min*^*-1*^*)*	*q*_*calc*_* (mg/g)*	*R*^*2*^	*k*_*2*_*(g/mg.min)*	*q*_*calc*_* (mg/g)*	*R*^*2*^	
Ba(II)	298 308 318 328	0.023 0.027 0.031 0.030	60.3 61.3 63.1 64.1	0.931 0.946 0.915 0.957	0.0005 0.0006 0.0007 0.0008	92.4 98.3 100.5 101.6	0.999 0.999 0.999 0.999	90.7 93.7 95.9 97.9
Cs(I)	298 308 318 328	0.005 0.006 0.007 0.007	114.11 117.25 118.45 119.44	0.989 0.987 0.941 0.951	0.0001 0.0002 0.0003 0.0004	79.2 80.5 85.1 94.1	0.989 0.999 0.999 0.999	76.3 80.3 84.4 87.7

##### Pseudo- second-order order

It is clear that, from the results inside Table 1, the pseudo-second—kinetic—model applied to Cs(I) and Ba(II) adsorption compared with the pseudo-first -model, the q_cal_ values estimated in the pseudo-second—model agreed well with the q_exp_ values, and also higher values of R^2^. The obtained values of q_cal_ and a great value of R^2^ illustrate that the mechanism adsorption process of the second model is prevalent, and the rate constant is controlled via the chemisorption mechanism^40^.

### Adsorption isotherms

**Figure 8:**
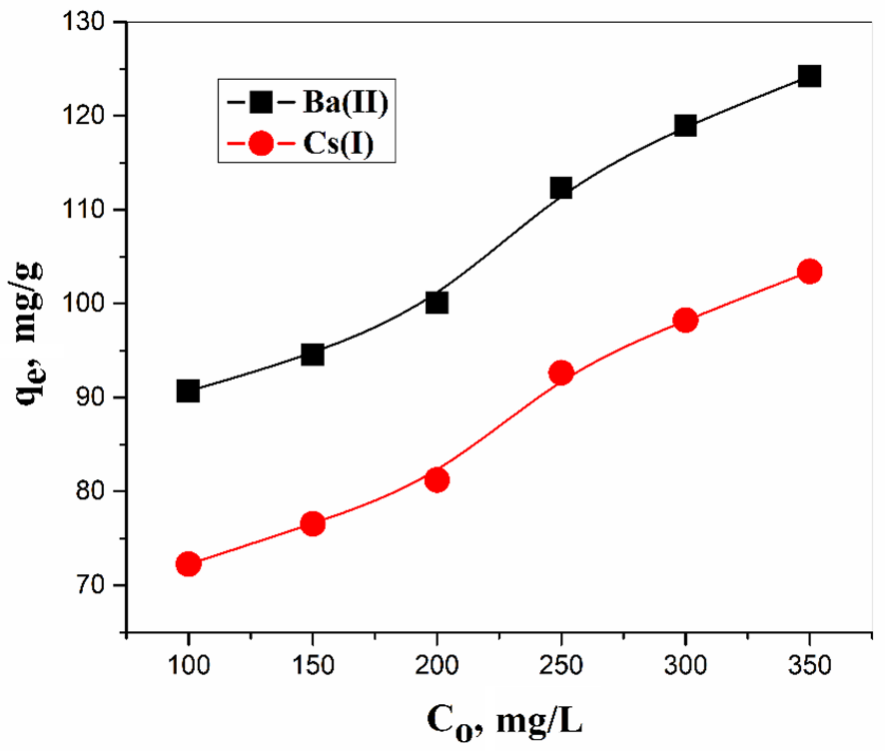
Effect of initial concentration on Ba(II) and Cs(I) adsorption.

The isotherm models generally give information about adsorption. Several parameters obtained from these models signalize the properties of the adsorbent at a certain condition from pH and temperature values. Isotherm studies have been illustrating the relationship between the concentration of ions (Ce) with the amount adsorbed onto ZFO/HA nanocomposite at equilibrium (q_e_)^41^. Figure 8 offers the relationship between the amount of Ba(II) and Cs(I) adsorbed at equilibrium q_e_ onto the zinc ferrite-humic acid nanocomposite with the initial metal ion concentrations Co. From the figure; it is clear that the adsorbed amount increase with the increase in the initial concentration of the ions. The values of q_e_ have been increased from 90.71 to 124.23 mg/g for Ba(II) while increased from 72.27 to 103.43 mg/g for Cs(I) onto zinc ferrite-humic acid nanocomposite.

**Figure 9:**
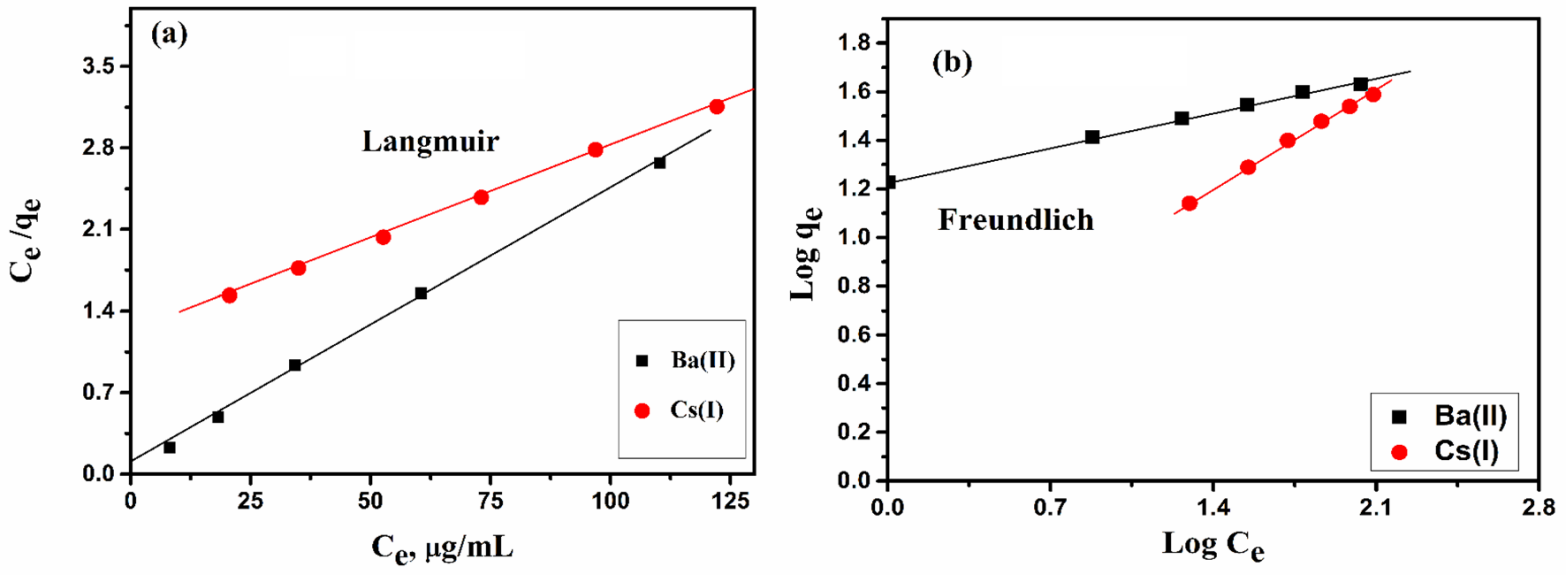
Isotherm plots for (**a**) Langmuir and (**b**) Freundlich models of Ba(II) and Cs(I) by the ZFO/HA nanocomposite.

The adsorption isotherm studies such as Langmuir and Freundlich were utilized for fit to obtained data, Fig. 9. Different isotherm parameters and R^2^ for these models are displayed in Table 2. It is the possibility to compute the parameters of these models from the linear relation, and the values of these parameters are displayed in Table 2. From obtained results, the two models showed an excellent simulation for the adsorption process to cesium and barium ions using different concentrations. Furthermore, the values of R^2^ were higher in the Langmuir model than in the Freundlich model.

**Table 2:** Isotherm parameters of adsorption process for **Ba(II)** and **Cs(I)** onto ZFO/HA nanocomposite.

Ion	Langmuir constants			Freundlich constants			*R*^*2*^
	*Q*_*max*_* (mg/g)*	*R*_*Ld*_	*b (l/mg)*	*R*^*2*^	*K*_*f*_* (mg/g)*	*n*	
Ba(II)	63.33	0.15	0.411	0.998	21.21	5.21	0.965
Cs(I)	42.55	0.018	0.124	0.998	5.33	2.43	0.976

The acquired parameters of the two models have been applied to compare the capacity of the ZFO/HA nanocomposite towards Ba(II) and Cs(I). Monolayer capacity, Q_max_, was defined from the straight line of the Langmuir model. Its values recorded 42.55 and 63.33 mg/g of Cs(I) and Ba (II), respectively. R_L_ values of Ba(II) and Cs (I) adsorption were illustrated in Table 2. These data signalize highly favorable adsorption (0 < R_L_ < 1)^37^. The values of n assess the intensity of the adsorption process, also the heterogeneous nature of the adsorbent surface. The closer the value of n from zero, the more heterogeneity of the adsorbent surface was expected; as n values are greater than one, necessitate chemical adsorption^11^.

### Thermodynamic studies

**Figure 10:**
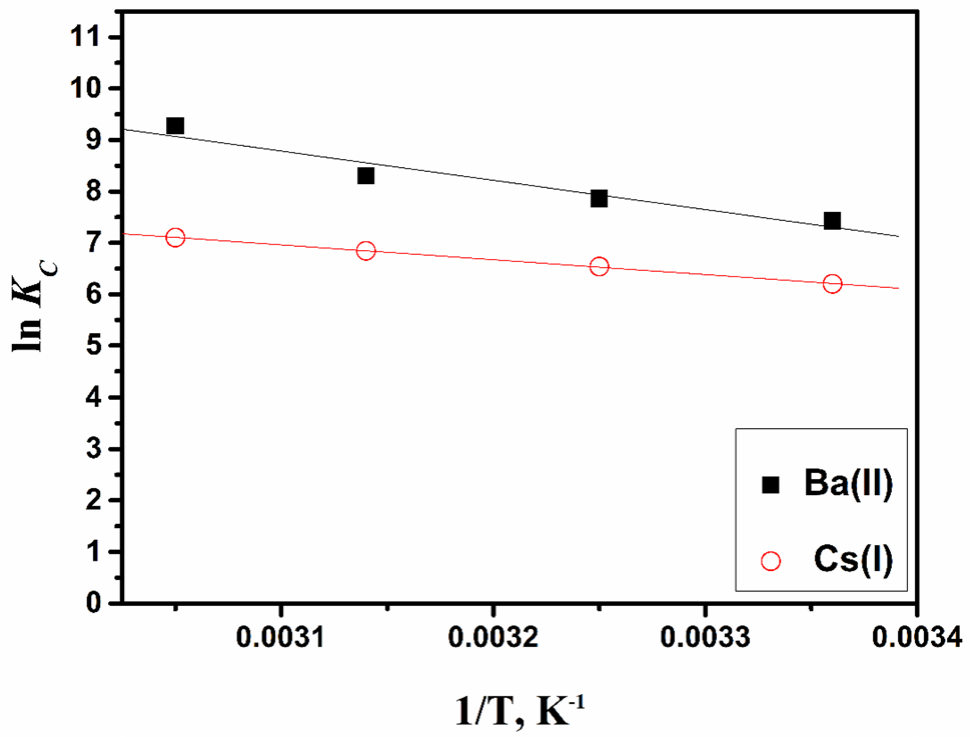
Van’t Hoff plots for the adsorption of Ba(II) and Cs(I) by ZFO/HA nanocomposite.

To assess thermodynamic constants for the adsorption, several parameters were assessed. Gibb’s free energy, ΔG°, is an essential norm for spontaneity. The process occurs spontaneously at a certain temperature when the ΔG° values equal negative values. Serval thermodynamic parameters, such as ΔG°, ΔH° (enthalpy change) as well as ΔS° (entropy change), were determined. Different values of ΔH°, as well as ΔS°, are gained from linear relation (ln Kc with 1/T), Fig. 10.

The different calculated thermodynamic parameters for the cesium and barium are illustrated in Table 3. The values for ΔG° are negative; this signalizes the spontaneous adsorption nature of cesium and barium onto the ZFO/HA nanocomposite. Also, the values of ΔH° are positive, indicating the endothermic nature of the adsorption of cesium and barium, while the values of ΔS° are positive. This displays the increasing randomness at the zinc/liquid interface through the adsorption of ions onto ZFO/HA nanocomposite^12,42,43^.

**Table 3:** Thermodynamic parameters for the adsorption of **Ba(II)** and **Cs(I)** by ZFO/HA nanocomposite.

Ion	Temp., (K)	∆H° (KJ/mol)	∆S° (J/mol.K)	∆G° (KJ/mol)
Ba(II)	298 308 318 328	52.22	234.35	− 20.22 − 22.12 − 23.55 − 24.34
Cs(I)	298 308 318 328	19.77	119.68	− 13.55 − 15.44 − 17.43 − 18.23

### Separation studies

The Ba(II) and Cs(I) separation factors can be calculated at different pH values using Eq. (3) and the data displayed in Table 4. The pH values variation study was performed in the range of 1–5, and the maximum separation between two ions was achieved at pH 5 (α_Cs/Ba_ = 3.3). Also, the highest separation factor was attained using a V/m ratio equal to 0.01 L/g (α_Cs/Ba_ = 2.6), as shown in Table 4.

**Table 4:** Impact of pH and V/m values on the separation of Ba(II) and Cs(I) by ZFO/HA nanocomposite.

pH	Separation factor	V/m	Separation factor
1	1.5	0.5	1.9
2	2.2	0. 3	1.4
3	2.4	0.1	2.4
4	2.6	0.03	2.1
5	3.3	0.01	2.6

### Desorption Studies

Various reagents have been applied to recover Ba(II) and Cs(I) from ZFO/HA nanocomposite, such as hydrochloric acid, sulfuric acid, nitric acid, and sodium hydroxide. The desorption experiments were achieved in an aqueous solution of HCl and H_2_SO_4_; it is clear that the recovery of Ba(II) and Cs(I) was insignificant. This can be attributed to the intense binding between Ba(II) and Cs(I) on ZFO/HA nanocomposite. However, the recovery of Cs(I) and Ba(II) can be achieved at 1.0 M HNO_3_ (80.2 and 35%), respectively. On the other hand, the recovery of Ba(II) and Cs(I) in 1.0 M NaOH is 60.5 and 62.2%, respectively, as shown in Table 5.

**Table 5:** Desorption of Ba(II) and Cs(I) onto ZFO/HA nanocomposite using different reagents.

Reagent	Concentration, M	Desorption percent, %	
		Ba(II)	Cs(I)
HNO_3_	1.0	35	80.2
HCl	1.0	0	0
H_2_SO_4_	1.0	0	0
NaOH	1.0	60.5	62.2

### Comparison with other adsorbents

The adsorption capacity of ZFO/HA nanocomposite for adsorption of Ba(II) and Cs (I) from aqueous solution was compared with other materials reported in the literature and is shown in Table 6. The results revealed that the ZFO/HA nanocomposite exhibited a higher adsorption capacity than other adsorbents, as reported in the literature. The results also indicated that the ZFO/HA nanocomposite could be a promising sorbent material for removing Ba(II) and Cs (I).

**Table 6:** Comparison of *Q*_*max*_ for **Ba(II)** and **Cs(I)** adsorption onto ZFO/HA nanocomposite with other materials.

Material	Adsorption capacity (mg/g)		Reference
	Ba(II)	Cs(I)	
Allophane adsorbent	38.2	NR	^44^
Spherical ZnO	64.6	NR	^45^
Modified Salvadora Persica	NR	200	^46^
Magnetic graphene oxide	NR	9.29	^47^
Activated charcoal modified	NR	63.1	^48^
Ceric oxide	0.051	NR	^49^
spent coffee waste	6.17	NR	^4^
Dolomite powder	3.9	NR	^50^
Nickel hexacyano ferrate incorporated walnut shell	NR	4.94	^51^
Copper hexacyanoferrate–PAN composite Black carbon	NR	25.45	^52^
zinc ferrite-humic acid composite	63.33	42.55	Present work

*NR* not reported.

## Conclusions

Herein, we successfully synthesized a zinc ferrite-humic acid nanocomposite. The prepared zinc ferrite-humic acid (ZFO/HA) nanocomposite was well characterized using different techniques. The XRD and FTIR studies confirmed the successful ZFO/HA nanocomposite preparation. SEM images illustrated that the ZFO/HA nanocomposite has a flake-like shape. Also, EDX spectra showed the purity of the ZFO/HA nanocomposite. The optimum conditions for adsorption of Ba(II) and Cs(I) onto zinc ferrite-humic acid composite were achieved as pH 5, and equilibrium time was 100 min and 150 min for Ba(II) Cs(I) as well as initial concentration was 100 mg/L, and V/m ratio was 1 L/g. The ZFO/HA nanocomposite adsorption capacities were 62.33 mg/g for Ba(II) and 42.55 mg/g for Cs(I). The adsorption process fits well with a Langmuir adsorption model. The thermodynamic data indicate that adsorption is exothermic and spontaneous, and an increase in randomness in the system. Ba(II) and Cs(I) have the highest separation factors in acidic solutions at pH 5.0 and a V/m ratio of 0.01 L/g. Desorption tests employing 1.0 mol/L nitric acid revealed maximum desorbed rates of 80.2% and 35.0% for Cs(I) and Ba (II), respectively. The study showed that the synthetic zinc ferrite-humic acid nanocomposite could be a promising exchange material for the Ba(II) and Cs(I) adsorption from wastewater.

## Data Availability

All data generated or analysed during this study are included in this published article [and its supplementary information files].
